# CoViD-19 Susceptibility

**DOI:** 10.6026/97320630016501

**Published:** 2020-07-31

**Authors:** Francesco Chiappelli

**Affiliations:** 1 Francesco Chiappelli, Ph.D., Dr. Endo (h.c), Professor Emeritus, UCLA, Center for the Health Sciences; CSUN, Department of the Health Sciences

**Keywords:** COVID-19, susceptibility, immunity

## Abstract

There have been over five million cases of infection with the second Corona virus to induce SARS (SARS-CoV2) and close to half a million deaths worldwide since the first report of Corona Virus Disease in late December 2019 (CoViD-19). Over two million CoViD-19 patients have recovered. The factors and variables that lead certain CoViD-19 patients to survive this otherwise aggressive and lethal viral infection are intensely researched, as is the development of productive anti-virals and of safe and effective vaccines. Several hypotheses invoke putative mutations of the ss-positive RNA SARS-CoV2 virus to states of stronger or weaker virulence and lethality. Other hypotheses propose that the patient's status of immunity, vitamin D level, Zinc deficiency or other physiological parameters determine how any given patient will effectively weather the viremia and the consequential multi-symptomatic CoViD-19. The initial cause - causa prima - underlying all the symptoms of CoViD-19 is infection of the host human cell by SARS-CoV2. The virus spike (S) protein finds its binding site, ACE2, widely distributed in all cells and tissues that potentially proffer CoViD-19 pathology. S consists of two subunits, S1 and S2, which are cleaved by the widely expressed transmembrane protease serine 2 (TMPRSS2) before the virus fuses to the plasma membrane and infects the cell. Current trends show that variant alleles resulting from single nucleotide polymorphisms (SNPs) of ACE2, and genetic variants of TMPRSS2, with putative distinct affinities for S clip, may determine a complex multi-factorial spectrum of SARS-CoV2 virulence across patients, and predict CoViD-19 susceptibility.

## Background

CoViD-19 is a complex disease with a spectrum of signs and symptoms, from initial fever, confusion, chills, fatigue, generalized myalgia, malaise, drowsiness, cough, to acute respiratory distress syndrome with dyspnea and pneumonia, severe gastrointestinal disorder with diarrhea, liver injury, coagulation dysfunction, cardiovascular failure, loss of taste and smell and other clinically relevant neurological involvement. Although early statistics indicated that CoVid-19 predominantly afflicted the aging population, recent data show that young adults are at risk as well, and that children may develop a pathological profile akin to Kawasaki's pediatric inflammatory condition and to the Multisystem Inflammatory Syndrome in Children (MIS-C). The broad spectrum of CoViD-19 across gender, ethnic and age groups derive from, and are related to a severe unregulated cytokine storm, consequential to infection with SARS-CoV2. Therefore, it was proposed that CoViD-19 might be best named Corona Virus Syndrome 2019 (CoViS-19) [1].

SARS-Cov2 is a positive-sense single-stranded RNA virus (+ssRNA; 29,903 bases - NCBI genome ID: MN908947). Of its four structural proteins, S (spike), E (envelope), M (membrane), and N (nucleocapsid), and other minor proteins, the virus negotiates its binding, adhesion, fusion and penetration into the host cells via the two glycosylated subunits of S. Glycoprotein S subunit 1 (S1) binds to, and blunts the expopeptidase activity of dipeptidyl peptidase-4 (adenosine deaminase complexing protein-2, CD26), an important member of the Peptidase Targeted Immuno-regulation (PeTIr) membrane-associated enzyme family. The S1/S2 adhesion site is similar in structure to the staphylococcal enterotoxins B super-antigen motif [2], stretches of microbial peptides that can polyclonally activate up to 20% of T cells, and escape normal antigen processing by antigen presenting cells by binding directly to the T cell receptor. The S1/S2 interface may contribute in that manner to the generation of exhausted T cells (Tex), and to the loss of regulatory T cells (Treg) [3].

The S1/S2 complex attaches to the host cell membrane via the single-spanning transmembrane Zinc-dependent angiotensin-converting enzyme-2 (ACE2), a single-pass type-I 805 amino-acid long protein, endowed with functional N-terminal peptidase M2 domain (residues 19-615) and a C‐terminal collectrin renal amino acid transporter domain (residues 616-768) that includes a ferredoxin-like fold 'neck' domain (residues 615-726) ending with an hydrophobic transmembrane hydrophobic helix region of 22 amino acids followed by an intracellular segment of 43 amino acids. ACE2 ubiquitous expression in most organ cells, from lung alveolar epithelial cells to small intestine enterocytes, vascular endothelial cells and neural cells, informs the wide infection span of SARS-Cov2. The bound complex is primed by widely expressed transmembrane protease serine-2 (TMPRSS2), which clips S2 to expose the viral fusion peptide that permits SARS-CoV2 viral entry into the cell [1,4].

The S1/S2 unit also binds to CD147 (i.e., basigin; Extracellular Matrix MetalloProteinase Inducer, EMMPRIN),a ubiquitous extra-cellular membrane-bound Ig-superfamily metallo-protease inducer. CD147- S1/S2 interaction may be involved in SARS-Cov2 host cell invasion [1,5,6]. The physiological role of CD147 pertains to the inflammation and proteolysis process in the context of the initial and progression of atherosclerotic lesions. Single-nucleotide polymorphisms (SNPs) of the CD147 gene, including rs8259T/A in the 3'-untranslated region, preferentially increase patient susceptibility to acute coronary syndrome [7].

SNPs refer to substitutions of a single nucleotide that occur at specific positions of a gene, where each variation is present at a level of 0.5% from person to person in the population. SNPs pinpoint differences in susceptibility to a wide range of diseases, variability in response to treatment interventions, phenotypic clues and relevant pharmaco-genomic as well as, putatively, vaccine targets.Non-coding SNPs, as CD147 rs8259, occur in non-coding regions of the genome, and manifest as increased risk of a specific disease, or as modifiers of the levels of expression of a given gene. Coding SNPs can alter the translated section of the gene either by synonymous substitutions (e.g., GGC codon to GGT, or vice versa, both encoding glycine), or nonsynonymous substitutions, which can result in either mis-sense or non-sense point mutations. Several human genes associated with viral infections contain SNPs [8,9].

Case in point, SNPs provide timely and critical insights about the pathology in human infection by influenza A virus, and the selective susceptibility of some individuals to suffer more severe symptoms, relative to others [8].SNPs can also point to novel and improved anti-viral immunotherapies, including in HIV/AIDS, IL-7R-a SNP rs6897932, involved in improved CD4+ T cell recovery following combination antiretroviral therapy [10].

In addition to the CD147 locus, SNPs of ACE2 and of TMPRSS2 seem critical in determining the susceptibility of individual patients to SARS-Cov2 infection, and to CoViD-19 severity. By X‐ray diffraction and cryo-electron microscopy, two of eleven characterized ACE2 SNPs, namely rs73635825 (S19P) and rs143936283 (E329G), vary substantially in their intermolecular interactions with the S1/S2 spike complex. These SNPs are characterized by low binding affinity for S, and by lacking certain residues key to the ACE2-S1/S2 spike complex formation [11], and may therefore lower CoViD-19 susceptibility.

Virions of the influenza virus family (Type A, B, C, D) are similar in structure, which consist, as for the Corona family, of a viral envelope containing two main types of proteins, wrapped around a central core. The hemagglutinin protein mediates binding of the virion to target cells and entry of the viral genome into the target cell. Cleavage of hemagglutinin by host cell proteases, essential for influenza virus infectivity, is, as for virions of the Corona virus family (e.g., SARS-Cov2), brought about by TMPRSS2 [12]. Whereas the field of research for SARS-Cov2 virulence is novel and emerging, genomic investigations of TMPRSS2 SNPs have indicated that genomic SNP rs2070788, a higher-expression variant of TMPRSS2 (Odds Ratio = 2.11, CI95: 1.18 to 3.77; p=0.01), and rs383510 are among the highest risk variant to severe A(H1N1)pdm09 and A(H7N9) influenza [13]. It is reasonable to expect that rs2070788 and rs383510 may also be high-risk genomic variants for SARS-Cov2 virulence, and may raise CoViD-19 susceptibility.

In conclusion, susceptibility to CoViD-19 depends upon coronavirus–host interactions, which are key to viral pathogenesis and will ultimately determine the pathogenic outcome of infection [14]. It is possible and even probable that, in SARS-Cov2-positive patients, distinct prognosis of consequential CoViD‐19 will be associated with SNPs in ACE2, TMPRSS2 or CD147, either individually or in combination with each other, and in combination with inherent SARS-Cov2 mutations resulting into more or less virulent strains. Greater understanding of the interactions of the SNPs from these three genomic product to SARS-Cov2 binding and fusing to the host cell membrane should help identify high-risk individuals for better prophylactic and therapeutic intervention for CoViD-19.

To be clear, susceptibility to the multi-factorial pathology in CoViD-19 need not be restricted to ACE2, CD147 or TMPRSS2 polymorphism. Case in point, a peer-reviewed pre-print report shows that the e4e4 SNP of the 35 kDa lipid-transport glycoprotein apolipoprotein E, encoded by the APOE gene and associated with dementia of the Alzheimer's type [15], at least doubles CoViD-19 susceptibility risk (Odds Ratio = 2.31, CI95: 1.65 to 3.24, p=0.000001) [16].

## References

[1] Chiappelli F (2020). Bioinformation.

[2] Chiappelli F (2020). Bioinformation.

[3] Chiappelli F (2020). Bioinformation.

[4] Hoffmann M (2020). Cell.

[5] Chen Z (2005). J Infect Dis..

[6] Wang K (2020). BioRxiv.

[7] Yan J (2015). Medicine.

[8] Nogales A (2019). deDiego Pathogens..

[9] Zhang Z (2019). Front Genet..

[10] Resino S (2019). Biomolecules..

[11] Hussain M (2020). J Med Virol..

[12] Limburg H (2019). J Virol..

[13] Cheng Z (2015). J Infect Dis..

[14] de Wiide AH (2018). Roles of Host Gene and Non-coding RNA Expression in Virus Infection..

[15] Yu JT (2014). Annu Rev Neurosci..

[16] https://academic.oup.com/biomedgerontology/advance-article-abstract/doi/10.1093/gerona/glaa131/584345.

